# Gynecologic and Reproductive Health in Patients with Sickle Cell Disease: Underrecognized Challenges and Care Management Opportunities

**DOI:** 10.3390/jcm15030923

**Published:** 2026-01-23

**Authors:** Rachel Michel, Caitlin S. Stukel, Alexandra Myers, Abigail Lepsch Combs, Mostafa A. Borahay, Alexander D. Fuld, Gregory W. Kirschen

**Affiliations:** 1 Geisel School of Medicine, Dartmouth College, Hanover, NH 03755, USA; rachel.michel.med@dartmouth.edu (R.M.); caitlin.s.stukel.med@dartmouth.edu (C.S.S.); alexandra.v.myers.med@dartmouth.edu (A.M.); alexander.d.fuld@dartmouth.edu (A.D.F.); 2 Department of Gynecology & Obstetrics, Johns Hopkins University, Baltimore, MD 21287, USA; acombs6@jh.edu (A.L.C.); mboraha1@jhmi.edu (M.A.B.); 3 Section of Hematology and Oncology, Dartmouth Hitchcock Medical Center, Lebanon, NH 03755, USA; 4 Department of Obstetrics & Gynecology, Hospital of the University of Pennsylvania, Philadelphia, PA 19104, USA

**Keywords:** diminished ovarian reserve, infertility, recurrent pregnancy loss, genetic counseling, reproductive autonomy

## Abstract

Sickle cell disease is an autosomal recessive hemoglobin disorder affecting about 100,000 people in the United States, predominantly those of African descent. A point mutation in the β-globin gene in red blood cells causes these cells to sickle under hypoxemic conditions, reducing blood flow and oxygen delivery to tissues. This manifests in the form of painful vaso-occlusive episodes, acute chest syndrome, and acute infarction of various organs, including the spleen, bone, and lung. While sickle cell disease complications such as hemolytic anemia, tissue hypoxia, and chronic organ damage are well studied, attention to the unique reproductive challenges faced by patients with sickle cell disease remains underrecognized and underappreciated. This review aims to explore key reproductive health issues in patients with sickle cell disease, including diminished ovarian reserve, infertility, and obstetric and perinatal risk. Secondly, this review aims to identify key counseling and care opportunities for providers to support patients with sickle cell disease in meeting their reproductive goals.

## 1. Introduction

**Figure 1:**
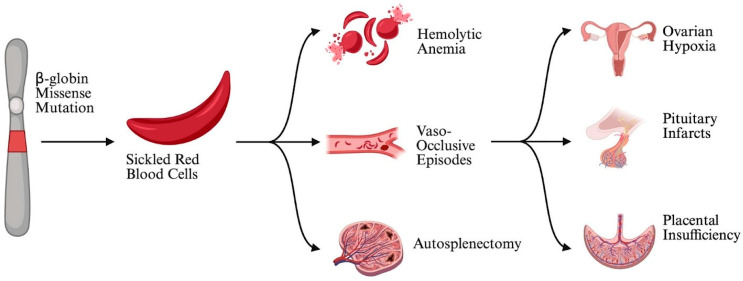
Pathophysiological cascade linking sickle cell disease genetics to adverse reproductive outcomes.

Sickle cell disease (SCD) is an autosomal recessive hematologic condition primarily affecting African Americans in the United States and globally affecting people of African, Middle Eastern, and South Asian descent [1]. An estimated 100,000 individuals are affected in the United States [2,3]. SCD is caused by a point mutation in the gene coding for the β-globin chain of hemoglobin, the multi-subunit protein in red blood cells responsible for carrying oxygen in the body [4]. This mutation alters the shape of red blood cells (RBCs), diminishing their ability to carry oxygen and obstructing circulation. This process, known as “sickling,” results in episodes of acute, severe pain known as sickle cell crises or vaso-occlusive episodes [4]. Patients with SCD thus experience increased rates of hemolytic anemia, vascular dysfunction, chronic organ injury, and tissue hypoxia [5,6] (Figure 1).

Treatment of acute vaso-occlusive episodes is focused on supportive care and includes analgesic administration, intravenous fluids, supplemental oxygen, and blood transfusions [4]. In addition, hydroxyurea, approved by the FDA in 1998, has demonstrated a reduced incidence of vaso-occlusive episodes and prolongation of life expectancy in individuals with SCD. Hydroxyurea upregulates fetal hemoglobin (HbF) levels in patients, improving oxygen delivery and reducing pain episodes and the need for frequent blood transfusions [7]. Mechanistically, hydroxyurea is a ribonucleotide reductase inhibitor that promotes stress erythropoiesis, thereby increasing HbF synthesis in erythroid precursors. Elevated HbF within red cells interferes with HbS polymerization, resulting in less rigid, more deformable erythrocytes and thus mitigates anemia [8]. Allogeneic hematopoietic stem-cell transplantation (HSCT) from an HLA-matched donor is a proven curative therapy for sickle cell disease. However, its use is limited by donor availability and transplant-related risks. In 2023, two autologous gene-based therapies were approved by the FDA for patients 12 years and older with a history of severe vaso-occlusive complications. The long-term efficacy and potential risks of gene-based therapy are not yet fully known, and issues of access and cost remain concerns.

With improved management and treatment of SCD, the formerly life-threatening pediatric hematological disorder has evolved into a chronic disease of adults. Consequently, parenthood has now become a realistic and important goal for many patients. While the majority of pregnancies in patients with SCD result in the delivery of a healthy neonate, this population experiences a markedly elevated risk of endocrine dysfunction, infertility, and obstetric complications [9]. Quantifying reproductive risk for patients with SCD is difficult because outcomes such as miscarriage, subfertility, and early pregnancy loss are often under-reported or misclassified. These complications may be due to disease pathology, treatment side effects, or a combination of both [10]. Thus, this review aims to explore key reproductive challenges in patients with SCD, including diminished ovarian reserve, infertility, and increased obstetric and perinatal risk, exploring both disease and treatment-related pathology. Secondly, this review aims to identify key counseling and care opportunities for providers to support patients with sickle cell disease in meeting their reproductive goals.

## 2. Micro-Infarction, Inflammation, and Infertility

Infertility significantly contributes to the overall reproductive burden experienced by patients with SCD. Infertility affects 23.9% of women with SCD, compared to 11% of women in the general US population [11]. In addition, compared to the general population, patients with SCD are reported to have diminished ovarian reserve (DOR) [12,13]. The pathogenesis of SCD may contribute to DOR and may affect oocyte quality. Folliculogenesis in the ovaries is a tightly regulated process that depends on a well-oxygenated microvascular supply [14]. Thus, excessive hypoxia from vaso-occlusive episodes in SCD can impact this process. Growing follicles are highly proliferative and therefore susceptible to sickle-induced micro-thrombi that block the capillaries feeding the follicle. In addition, once occlusion occurs in the ovarian microvasculature, there is limited alternative perfusion to offset the damage, increasing the risk of ischemic injury. Repeated micro-infarction can cumulatively deplete the primordial follicle pool and produce chronic inflammation and oxidative stress. Micro-infarcts and the downstream effects on the ovarian environment may contribute to diminished ovarian reserve and poor oocyte quality [12,14].

Patients with SCD may experience dysfunction of the hypothalamic-pituitary-ovarian (HPO) axis. Chronic anemia, energy imbalance, and the stress of chronic illness may contribute to HPO suppression and the menstrual irregularities reported by adolescents with SCD [15,16]. Furthermore, patients with SCD experience episodes that cause infarcts in the posterior pituitary. These infarcts may injure the gonadotrophs—the endocrine cells responsible for producing gonadotropin hormones—leading to decreased FSH and luteinizing hormone (LH), impacting ovarian function [17]. Similar to the ovarian environment, pro-inflammatory cytokines produced by sickled cells may contribute to HPO dysfunction. For example, one study found that monocytes from patients with SCD expressed increased IL-1 [18]. In rats, IL-1 has been demonstrated to inhibit the release of hypothalamic LH-releasing hormone [19]. Thus, it is possible that the upregulation of pro-inflammatory cytokines released by sickled cells disrupts endocrine function. Consequently, chronic micro-infarction and the resulting hypoxic and inflammatory environment may contribute to diminished oocyte quantity, quality, and function of the HPO axis, thus potentially explaining mechanisms of infertility and subfertility.

## 3. Obstetric and Perinatal Risk

Reproductive challenges for patients with SCD extend beyond conception. The multifaceted risks arise from the underlying pathophysiology of SCD, which is amplified by the stressors of pregnancy. Patients with SCD experience markedly elevated rates of miscarriage in the US (36% versus 21.3% in racially matched controls) [20,21]. In addition, recurrent pregnancy loss (≥two consecutive miscarriages) is reported in ~12% of patients with SCD, compared with only 2–3% in racially and age-matched controls [20,22]. Furthermore, fetal growth restriction, low birth weight, and perinatal mortality occur at an elevated frequency in patients with SCD compared to the general population [23,24]. Taken together, these data illustrate an elevated risk of fetal and neonatal morbidity and mortality in pregnancies complicated by SCD.

The increased metabolic demands and relative hypoxemia of pregnancy can precipitate sickling, especially during periods of stress, infection, dehydration, and especially around the time of delivery [25]. Sickled erythrocytes promote hemolysis, increased platelet activation, and a prothrombotic environment. Pregnancy further amplifies this hypercoagulable state by increasing clotting factor levels and reducing fibrinolysis. Thus, the intrinsic hypercoagulability of SCD is compounded by pregnancy, markedly raising the risk of vaso-occlusive episodes, thromboembolism, and acute chest syndrome [26,27]. These episodes often require hospitalization, pain management, and transfusion, and their frequency is a strong predictor of adverse maternal and fetal outcomes.

Sickling and vaso-occlusion can also occur within the placenta, leading to infarction, hemorrhage, and necrosis that contribute to placental insufficiency. Histopathology studies comparing placentae from patients with SCD to controls have demonstrated clear morphological differences, including increased syncytial knot formation, a marker of physiologic stress [28]. Resultant compromised placental function reduces the transplacental delivery of oxygen and nutrients to the fetus, creating a relatively hypoxic uteroplacental environment [29]. In response to chronic hypoxia, placental metabolic demand is reduced, and fetal growth is restricted [30]. Fetal growth restriction is associated with a markedly increased risk of preterm delivery and stillbirth. It is hypothesized to have long-term consequences for cardiovascular, metabolic, and neurological development extending into adulthood [31].

Another prominent feature of SCD is end-organ damage and failure. Pregnancy can exacerbate preexisting organ damage, including renal disease, pulmonary hypertension, and cardiac dysfunction. In addition, 35–70% of adults with SCD have either complete auto-splenectomy or severe splenic hypofunction, which puts the patient and fetus at heightened infection risk, especially from encapsulated organisms [32]. Altogether, patients with SCD face a higher incidence of adverse outcomes across the entire spectrum of conception, gestation, and delivery. 

## 4. Treatment Impact on Reproductive Outcomes

### 4.1. Hydroxyurea

In addition to SCD pathology, treatments may play a role in the complex reproductive issues patients face. Hydroxyurea is a commonly prescribed treatment for SCD patients that works by increasing the concentration of HbF, thereby decreasing the number of sickled cells in circulation [7]. While hydroxyurea has been shown to reduce vaso-occlusive episodes in patients with SCD, its potential impact on reproductive health is not well described. Data are inconclusive, with some studies demonstrating diminished ovarian reserve in patients with SCD taking hydroxyurea, thus potentially impacting fertility [33,34,35,36]. It is hypothesized that hydroxyurea may exhibit dose-dependent cytotoxicity, potentially harming oocytes in late-stage follicles, which are particularly sensitive to DNA damage [37]. Patients on hydroxyurea have been counseled to avoid pregnancy and are placed on contraception while on the drug, potentially delaying childbearing and increasing the risk of age-related infertility. For patients who do choose to discontinue Hydroxyurea, current guidelines suggest this be conducted at least 3 months prior to attempted conception [38].

Historically, hydroxyurea was considered contraindicated in pregnancy based on teratogenicity demonstrated in large animal studies at doses 5–10 times higher than therapeutic human doses [39,40,41]. The teratogenic effects of hydroxyurea are known to be dose- and species-dependent, yet hydroxyurea has been avoided during pregnancy largely due to these studies. Limited studies document prenatal hydroxyurea exposure, either as monotherapy or in combination with other agents, and findings are inconsistent, with small sample sizes [42]. Recent evidence suggests that the risks of untreated disease—particularly increased vaso-occlusive episodes—may outweigh potential hydroxyurea-related adverse effects [43]. Well-designed prospective studies with precise dosing and exposure data are needed to clarify the relationship between hydroxyurea dose, maternal-fetal exposure, and pregnancy outcomes.

### 4.2. Iron Overload

**Figure 2:**
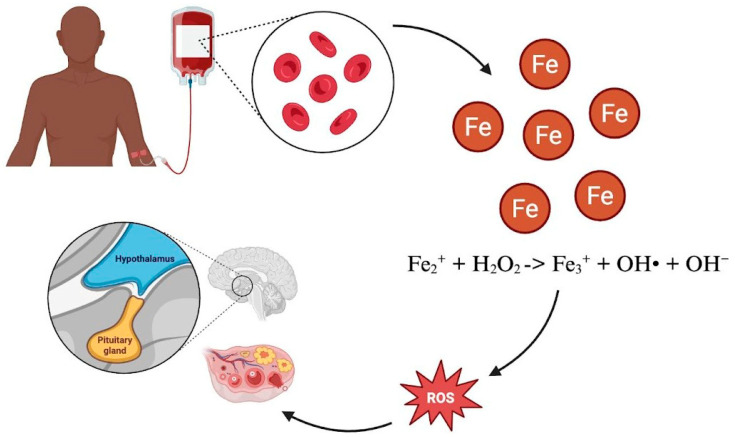
The body can accumulate excess iron from chronic red blood cell transfusions, which reacts with hydrogen peroxide (H_2_O_2_) to form hydroxyl radicals. These reactive oxygen species (ROS) can cause oxidative injury to the hypothalamus, pituitary gland, and ovaries.

Patients with SCD often require chronic red blood cell transfusions—both prophylactically and therapeutically—to reduce concentrations of sickled red cells and improve oxygen delivery to tissues [44]. However, the human body does not have a mechanism for excreting excess iron; therefore, chronic transfusions pose a risk of iron overload, which can, in turn, cause end-organ damage through oxidative injury [45]. With respect to reproductive function, iron overload from regular blood transfusions has been associated with diminished ovarian reserve, oocyte maldevelopment, and dysfunction of the HPO (Figure 2) [10,46,47]. Repeated transfusions further increase the risk of development of maternal erythrocyte alloantibodies, which can cross the placenta during gestation and can lead to alloimmunization, fetal anemia through red blood cell destruction, and ultimately hydrops fetalis and fetal demise [48].

While chelation therapy is a treatment option to remove excess iron, its clinical use is impacted by factors like low adherence, side effects such as visual and auditory neurotoxicity, high cost, and limited efficacy [45,49,50]. In addition, chelation therapy can remove iron buildup but cannot undo tissue damage or endocrine dysfunction caused by hormonal axis suppression.

### 4.3. Hematopoietic Stem-Cell Transplantation

Hematopoietic stem-cell transplantation (HSCT) remains the primary curative treatment for patients with SCD, yielding event-free survival and cure rates exceeding 90% [51]. However, HSCT poses a serious risk of primary ovarian insufficiency (POI) from the use of gonadotoxic agents and total body irradiation. Typical pre-transplant doses of alkylating agents and irradiation yield complete destruction of the ovarian reserve, leading to POI in over 90% of post-pubertal patients [52,53]. As HSCT becomes more common, efforts to improve awareness and accessibility of fertility preservation should increase. Furthermore, reduced-intensity conditioning should be used where appropriate. Pregnancy is possible and safe in patients treated with HSCT; however, the rate of unassisted conception is quite low, underscoring the importance of fertility preservation in these patients’ family building.

### 4.4. Opioids

Opioids are a cornerstone of treatment for patients with SCD experiencing the excruciating pain of vaso-occlusive episodes. However, recurrent use becomes considerably more complex during conception and pregnancy. Opiate receptors are present throughout the reproductive system and are critical in the normal function of the HPO. Long-term opioid use has been associated with decreased secretion of Gonadotropin-releasing hormone (GnRH), in turn leading to reduced levels of LH, impairing ovulation [54]. Furthermore, mouse studies demonstrate that administration of morphine can interact with endogenous opiate receptors in the embryo and in the endometrium, which can interfere with successful blastocyst development and implantation [55].

If conception is successful and pregnancy is maintained, extensive or prolonged prenatal opioid exposure has been associated with several adverse obstetric and neonatal outcomes, most notably fetal growth restriction, neonatal opioid withdrawal syndrome, and long-term developmental delays [56]. Current literature further suggests that these risks are dose-dependent, underscoring the clinical importance of minimizing both the frequency of vaso-occlusive episodes and cumulative opioid exposure. Although opioids remain essential for analgesia in this population, especially given the contraindication to nonsteroidal anti-inflammatory drugs (NSAIDs) during pregnancy, discussions around their impact on endocrine function, fertility, and obstetric outcomes are essential.

## 5. Counseling and Care Opportunities for Providers

SCD remains markedly under-investigated, a shortfall that reflects its disproportionate prevalence among people of African ancestry [2,3]. This demographic concentration helps explain the persistent lacunae in the scientific literature and clinical guidelines surrounding the disease. When the disease intersects reproductive health—a domain that is itself understudied—the evidence base shrinks even further, leaving clinicians without robust guidance. As SCD becomes more manageable with advancements in therapies, our focus can shift from simply ensuring patients survive to reproductive age to actively supporting their ability to meet reproductive goals [57]. In the face of these dual knowledge gaps, physicians must adopt a proactive, transparent stance: they should initiate frank, empathetic conversations with patients, clearly delineating what is known, what remains uncertain, and how best to navigate care decisions in the interim.

### 5.1. Longitudinal Reproductive Counseling

It is essential that clinicians initiate age-appropriate and longitudinal discussions with patients about how sickle cell disease may affect family planning, fertility, and the health of future offspring. These conversations should begin before a patient contemplates conception, allowing sufficient time for shared decision-making. Rather than imposing a directive that patients with SCD avoid pregnancy, providers should frame the dialogue around realistic expectations for a pregnancy complicated by SCD and empower patients to weigh the information, voice their values, and co-create a personalized reproductive-health plan [58].

Initiating early, candid conversations about family planning and fertility with patients before beginning treatment empowers them to weigh the risks and make informed decisions about their future reproductive health. Guiding the patient and their family through fertility sparing options in early diagnosis of SCD can be a fruitful discussion, but one requiring age-appropriate language and sensitivity. For patients undergoing HSCT, consideration should be given to fertility preservation prior to therapy, whether through ovarian tissue cryopreservation for the pre-pubescent patients or oocyte cryopreservation for postmenarchal patients. The physical, psychological, ethical, and financial impact of these difficult circumstances cannot be overstated, and multi-disciplinary care between Hematology/Oncology, Reproductive Endocrinology and Infertility, Maternal–Fetal Medicine, Pediatrics, and Behavioral Health/social work teams is critical [10,59]. Furthermore, insurance coverage for fertility preservation and infertility treatment should be expanded to improve access to appropriate care.

### 5.2. Genetic Implications for Offspring

**Figure 3:**
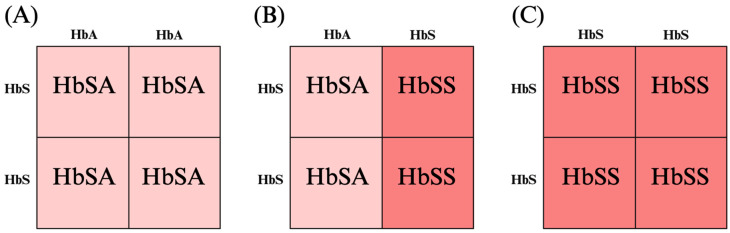
Genetics of sickle cell disease. (**A**) If one parent has sickle cell disease (HbSS) and the other parent lacks any sickle cell pathogenic alleles (HbAA), then 100% of offspring will have sickle cell trait (HbSA). (**B**) If one parent has sickle cell disease (HbSS) and the other has sickle cell trait (HbSA), there is a 50% chance that the offspring will be born with sickle cell disease. (**C**) If both parents have sickle cell disease (HbSS), then 100% of offspring will have sickle cell disease (HbSS).

Clinicians should counsel patients on the genetics of sickle cell disease and explain the value of paternal genetic testing for accurate inheritance risk assessment. Because SCD is an autosomal recessive disorder, an offspring can inherit the disease only if both parents carry a pathogenic SCD allele (Figure 3). Consequently, if the father is confirmed not to be a carrier, the risk of SCD in the offspring is zero. If the patient has SCD and the partner has the trait, then there is a 50% chance that the offspring will be affected. If both parents have sickle cell trait, there is a 25% chance that the offspring will be affected. If the partner has SCD or sickle cell trait, preimplantation genetic testing (PGT) or invasive diagnostic testing with chorionic villus sampling or amniocentesis should be offered [60].

### 5.3. Pain Management During Pregnancy

Management of vaso-occlusive episodes during pregnancy should remain a top priority. Prior to conception, the risks of opioid use should be discussed while emphasizing that navigating a pregnancy without adequate pain management could actually be a greater risk to mother and fetus [61,62].

While more data on the safety of hydroxyurea use during pregnancy is needed, a definitive management plan regarding whether to continue or temporarily discontinue hydroxyurea should still be established for each patient in consultation with Hematology/Oncology and Maternal–Fetal Medicine. Clinicians should individualize recommendations based on each patient’s history of vaso-occlusive episodes. For individuals who experience occasional episodes (e.g., ≤1–2 episodes per year), a temporary discontinuation of hydroxyurea may be a reasonable and low-risk strategy. Conversely, patients with a high frequency of vaso-occlusive events (e.g., multiple episodes per year) face substantial maternal and fetal morbidity in the absence of medical management; in such cases, the potential dangers of uncontrolled sickle-cell disease may outweigh the still-uncertain teratogenic risk associated with continued hydroxyurea therapy. Ultimately, a shared decision-making process—grounded in a thorough discussion of the relative risks and benefits—is essential to arrive at a management plan that aligns with the patient’s values and clinical circumstances [62].

### 5.4. Vaccine Recommendations

Vaccinations are also an important consideration when discussing safe pregnancies. Since patients with SCD are often asplenic, particular emphasis should be placed on ensuring the patient is up to date with vaccinations against encapsulated organisms, including polyvalent pneumococcal, Haemophilus influenza type B, and meningococcal vaccines, in addition to standard immunizations recommended for all pregnant people [57]. While influenza, Haemophilus influenzae type B, and Hepatitis B vaccinations can all safely be administered during pregnancy, other recommended vaccines in the sickle cell population, including pneumococcal, meningococcal, MMR, varicella, and HPV, are considered contraindicated during pregnancy and should be administered ideally prior to conception, otherwise deferred until postpartum [63]. Ensuring the patient understands why they are at higher risk of these infections also facilitates compliance and establishes trust.

The management of SCD as it relates to reproductive freedom is underrepresented in the literature; thus, our goal has been to highlight the most pressing medical, ethical, and psychological issues this condition poses during the reproductive years.

## 6. Conclusions

As this review demonstrates, reproductive health complications in sickle cell disease represent a significant yet often overlooked dimension of patient care. By developing a comprehensive understanding of how SCD impacts fertility and pregnancy outcomes, healthcare providers can deliver patient-centered care that addresses the full spectrum of reproductive needs in this population. Future areas for investigation in this space include preserving pituitary/ovarian/uterine blood flow to improve fertility outcomes, strategies to enhance opioid-sparing pain management in the periconception and pregnancy periods, and the search for novel therapeutics to mitigate iron overload-induced gonadotoxicity.

## Data Availability

No new data were created or analyzed in this study.

## Abbreviations

The following abbreviations are used in this manuscript:

SCD	Sickle Cell Disease
RBCs	Red blood cells
HSCT	Hematopoietic stem-cell transplant
HbF	Fetal hemoglobin
DOR	Diminished ovarian reserve
HPO	Hypothalamic-pituitary-ovarian
LH	Luteinizing hormone
FSH	Follicle-stimulating hormone
ROS	Reactive oxygen species
POI	Primary ovarian insufficiency
GnRH	Gonadotropin-releasing hormone
NSAIDs	Nonsteroidal anti-inflammatory drugs
PGT	Preimplantation genetic testing
